# Anti-diabetic activity of aerial parts of *Sarcopoterium spinosum*

**DOI:** 10.1186/s12906-017-1860-7

**Published:** 2017-07-06

**Authors:** Uriel Elyasiyan, Adi Nudel, Nir Skalka, Konstantin Rozenberg, Elyashiv Drori, Rachela Oppenheimer, Zohar Kerem, Tovit Rosenzweig

**Affiliations:** 1 Department of Molecular Biology, Department of Nutritional Studies, Ariel University, 40700 Ariel, Israel; 2 Institute of Biochemistry, Food Science and Nutrition, The Robert H. Smith Faculty of Agriculture, Food and Environment, The Hebrew University of Jerusalem, Jerusalem, Israel; 3 Samaria and Jordan Rift R&D Center, 40700 Ariel, Israel; 4 Department of Chemical Engineering, Biotechnology and Materials, Ariel University, Ariel, Israel; 5 Diabest Botanical Drugs Ltd., 40700 Ariel, Israel

**Keywords:** Glucose uptake, Insulin signaling, Medicinal plant, *Sarcopoterium spinosum*, Type 2 diabetes

## Abstract

**Background:**

*Sarcopoterium spinosum* (*S. spinosum)* is used by Bedouin medicinal practitioners for the treatment of diabetes. While the anti-diabetic activity of *S. spinosum* root extract was validated in previous studies, the activity of aerial parts of the same plants has not been elucidated yet. The aim of this study was to clarify the glucose lowering properties of the aerial parts of the shrub.

**Methods:**

Anti-diabetic properties were evaluated by measuring the activity of carbohydrate digesting enzymes, glucose uptake into 3 T3-L1 adipocytes, and insulin secretion. Insulin signaling cascade was followed in L6 myotubes using Western blot and PathScan analysis.

**Results:**

Activity of α-amylase and α-glucosidase was inhibited by extracts of all *S. spinosum* organs. Basal and glucose-induced insulin secretion was measured in Min6 cells and found to be enhanced as well. Glucose uptake was induced by all *S. spinosum* extracts, with roots found to be the most effective and fruits the least.

The effect of *S. spinosum* on Akt phosphorylation was minor compared to insulin effect. However, GSK3β and PRAS40, which are downstream elements of the insulin cascade, were found to be highly phosphorylated by *S. spinosum* extracts. Inhibition of PI3K and Akt, but not AMPK and ERK, abrogated the induction of glucose uptake by the aerial parts of the shrub.

**Conclusion:**

The aerial organs of *S. spinosum* have anti-diabetic properties and may be used as a basis for the development of dietary supplements or to identify new agents for the treatment of type 2 diabetes.

## Background

Type 2 diabetes (T2D) is recognized as a global pandemic, affecting millions of people all over the world in both western and developing countries [1]. Rigorous glycemic control is important in order to reduce the risk of developing severe complications accompanying chronic hyperglycemia [2, 3]. Unfortunately, despite using anti-diabetic medications, a large number of patients fail to achieve the recommended treatment goals [4], emphasizing the importance of identifying new anti-diabetic agents that may support glycemic control.

The plant kingdom contains a huge number of bioactive compounds that might be used for various therapeutic purposes. Ethnopharmacology, which describes the use of plants by traditional medicinal practitioners, can be used to direct and optimize the search for such plant-based bioactive materials.

*Sarcopoterium spinosum (L.) Sp.* is a widely distributed chamaephyte of the Rosaceae family, growing in the eastern Mediterranean landscape. *Sarcopoterium spinosum* (*S. spinosum*) is mentioned as a medicinal shrub in a large number of ethnobotanical surveys, documenting the use of *S. spinosum* aqueous root extract by traditional medicinal practitioners for the treatment of diabetes, as well as for cancer therapy and pain relief [5–10].

In our previous studies, the anti-diabetic properties of root extract of *S. spinosum* was validated both in-vitro and in-vivo. Root extract facilitated glucose transport into target cells of insulin: hepatocytes, adipocytes, and myotubes. Lipolysis was inhibited by the extract and the signaling pathway of glycogen synthesis was activated [11]. All of these functions suggested that *S. spinosum* root extract has insulin-mimetic properties. In addition, insulin secretion was enhanced in-vitro, suggesting a stimulatory effect on pancreatic β-cells [11]. In-vivo studies revealed that the glucose lowering properties of *S. spinosum* are accompanied by lower serum insulin levels and improved insulin tolerance [11, 12], indicating that when *S. spinosum* is administrated to the animal, the improvement in insulin sensitivity enables a reduction, rather than a stimulation, of insulin release.

The anti-diabetic activities mentioned in the ethnobotanical literature [5, 6, 13–15], as well as in small experimental studies [16–18], are mostly attributed to the roots of *S. spinosum*, entailing a significant burden for the potential use of this shrub as a therapeutic agent. The need to uproot the shrub for the preparation of extract might lead to serious ecological and conservational concerns if one suggests harvesting wild plants [19]. In addition, if cultivated, it is postulated that a long growing period might be required in order to obtain shrubs that generate a sufficient amount of bioactive components [20], as demonstrated in other medicinal plants [21]. Thus, uprooting the shrub might create an economic burden for the development of a product based on *S. spinosum* roots. This obstacle is further intensified when taking into account the low seedling rate of *S. spinosum* [22].

There is limited evidence for the use of the whole plant for the preparation of an antidiabetic remedy rather than only the roots [8, 23]. Although activity extracted from a certain plant part suggests the presence of a similar activity from additional plant parts, many examples exist of herbs showing pharmacological properties in one, but not other, organs [24, 25], emphasizing the necessity of experimentally validating the activity of the aerial parts of *S. spinosum*. In this study, the antidiabetic properties of the fruits and leaf of *S. spinosum* were investigated for the first time, using several in-vitro models for the study of diabetes.

## Methods

### Chemicals, kits and reagents

IBMX, dexamethasone, insulin, 2-deoxy-d-glucose (2-DG), cytochalasin-B, α-amylase, α-glucosidase, dinitrosalicylic acid (DNS), p-nitrophenyl α-d-glucopyranoside (PNPG) and inhibitors of proteases and phosphatases were purchased from Sigma. BSA, reagents and media for cell cultures were obtained from Biological Industries (Beit Haemek, Israel). [^3^H]2-deoxy-d-glucose (1 mCi) and Optiphase scintillation solution were purchased from Perkin-Elmer. A CytoTox 96 assay kit was purchased from Promega. Cytochalasin B, LY294002, AKT inhibitor VIII and Compound C were purchased from CalBiochem. Anti-actin was obtained from MP Biomedicals. Other primary antibodies were obtained from Cell-Signaling Technology. Secondary antibodies were purchased from Jackson ImmunoResearch.

### Plant material and extract preparation

*Sarcopoterium spinosum (L.) Sp.* plants were collected from the wild in the area around Ariel University. The plants were identified by the botanical staff of the University*.* A voucher specimen of the plant was deposited in the Israel National Herbarium at the Hebrew University of Jerusalem (No. HUJ 102531). *S. spinosum* root, leaf, and fruit extracts were prepared according to the traditional method [9]. In 1 L of water, 100 g of fresh *S. spinosum* root, leaf or fruit (R/*S. spinosum*, L/*S. spinosum* or F/*S. spinosum*, respectively) were boiled for 30 min. The solutions were left at 4 °C overnight and the supernatants were transferred to a sterile bottle without disturbing the pellet, and kept at −20 °C.

### Polyphenol analysis

The levels of polyphenols in extracts were evaluated using the Folin-Ciocalteu method [26]. The extraction (0.1 ml) was added to 3 ml DDW and 0.25 mL of Folin-Ciocalteu Reagent (FCR). The mixture was allowed to equilibrate for 5 min and then mixed with 0.75 mL of 200 g/L sodium carbonate solution. After incubation at room temperature for 10 min, the absorbance of the mixture was read at 735 nm using the respective solvent as blank. The results were expressed as μg of catechin equivalents per mg dry material.

### RP-LC

RP-LC was performed using a Surveyor system (Thermo Finnigan) and Lichrospher 100 RP-18 5um column, at a flow rate of 1 ml/min. Gradient elution was performed using (A) Water + 0.01% TFA and (B) Acetonitrile + 0.01% TFA. Initial conditions were 100% A for 5 min, linearly changed to 95% A at 12 min, linearly changed to 90% A at 22 min, linearly changed to 85% A at 42 min and followed by a gradient to 100% B at 110 min. In addition, 150 μg/10 μl were injected and UV absorption was recorded at 224 nm.

### LDH release

Cytotoxicity was measured using a colorimetric assay kit (CytoTox 96Promega), measuring lactate dehydrogenase (LDH) release, and indicating cell lysis, based on the conversion of a tetrazolium salt into a red formazan product. 3 T3-L1 pre-adipocytes were cultured on a 96-well plate at a concentration of 5 × 10^4^ cells/ml in growth medium containing different concentrations of *S. spinosum* extracts. Following 24 h of incubation, LDH was measured in the culture supernatants according to the manufacturer’s instructions. For positive control, cells were incubated with lysis buffer 60 min prior to measurement. The dye intensity was measured by microplate reader (Tecan, Salzburg, Austria) at a wavelength of 490 nm.

### α-amylase and α-glucosidase inhibition assays

To determine α-amylase inhibition by *S. spinosum* extracts, the standard procedure [27] was performed. *S. spinosum* extracts (100 μl) were incubated with porcine pancreatic α-amylase (250 μl, 0.15 U/ml in phosphate buffer (20 mM), pH 6.7, containing 6.7 mM sodium chloride) for 20 min at 37 °C. Starch (250 μl, 0.5% *w*/*v*) was added and the mixtures were incubated for 15 min at 37 °C, followed by the addition of DNS (2 ml, DNS 40 mM, K^+^-Na^+^ tartrate 1 M, NaOH 0.4 M) and incubation at 100 °C for 10 min for color development. Optical density was measured at a wavelength of 540 nm.

To determine α-glucosidase inhibition, *S. spinosum* extracts (25 μl) were incubated with *Saccharomyces cerevisiae* α-glucosidase (25 μl, 0.5 U/ml) for 10 min at 37 °C, followed by the addition of p-nitrophenyl α-d-glucopyranoside (25 μl, 0.5 mM) and incubation at 37 °C for 30 min. Na_2_CO_3_ (100 μl, 0.2 M) was used to stop the reaction and optical density was measured at a wavelength of 405 nm.

The following formula was used to calculate α-amylase/α-glucosidase inhibition:

$$ Inhibition\ \left(\%\right)=\frac{OD\ (control)- OD(sample)\kern0.5em }{OD\ (conrtol)}\times 100 $$

Vmax and IC50 values were calculated using nonlinear regression, using the GraphPad Prism 5.0 software.

### Cell culture

3 T3-L1 pre-adipocytes were cultured and induced to differentiate as described before [12]. 3 T3-L1 adipocytes were used for experiments 14 days after the initiation of differentiation, when 80–90% of cells exhibited adipocyte morphology.

L6 myoblasts were grown in MEM-α containing 25 mM glucose, 10% FCS, 2 mM glutamine, and 1% ampicillin. Experiments were performed on differentiated myotubes. L6 differentiation was induced as described in our previous studies [12].

Min6 insulinoma cells were grown in DMEM containing 10% FCS, 2.25 g/L glucose, 10 mM HEPES, 1 mM sodium pyruvate, 2 mM glutamine, 64 μM β-mercaptoethanol and 1% ampicillin. All cells were grown at 37 °C in a humidified atmosphere containing 5% CO_2_.

### Glucose uptake

Differentiated adipocytes were preincubated for 2 h in serum-free DMEM, with or without the addition of LY294002 (25 μM) at the last 30 min, AKT inhibitor VIII (5 μM) or Compound C (10 μM) at the last 60 min or PD98059 (50 μM) at the last 15 min of starvation. Starvation media was replaced, and cells were treated for 30 min with *S. spinosum* extracts at the indicated doses or 100 nM insulin, in the presence or absence of the inhibitors. Cells were washed twice with PBS, followed by 5 min incubation in 37 °C in PBS solution containing 0.1 mM 2DG and 0.5 μCi [^3^H]-2DG. Non-specific glucose uptake was measured by the addition of 20 μM Cytochalasin-B to the 2-DG solution. Cells were then washed 3 times with cold PBS, and incubated in 1% SDS for additional 5 min in order to lyse the cells. The contents of each well were transferred to a different 96 plate containing Optiphase scintillation liquid, and counted using a MicroBeta counter (Perkin-Elmer).

### Preparation of cell lysates

Differentiated L6 myotubes were treated with 100 nM insulin or *S. spinosum* extract (root extract at 1 mg/ml; leaf and fruit extract at 2 mg/ml) for the indicated times. For the preparation of whole cell lysate, cells were washed with Ca^+2^/Mg^+2^-free PBS and then mechanically detached in RIPA extraction buffer supplemented with protease and phosphatase inhibitors. The samples were homogenized and centrifuged at 14,000 rpm for 20 min, and supernatant was collected. Protein concentration was measured using the Bradford method.

### PathScan analysis

Path Scan Akt Signaling Antibody Array (Cell Signaling) enables the simultaneous measurement of the phosphorylation of 16 proteins involved in Akt pathway. L6 myotubes were treated with various *S. spinosum* extracts (R/*S. spinosum* at 1 mg/ml, L/*S. spinosum* and F/*S. spinosum* at 2 mg/ml) for 20 min. Protein was extracted as described, and the assay was performed according to the manufacturer’s instructions. The results were immunodetected using the enhanced chemiluminescence method and were visualized by ImageQuant LAS4000 MINI (GE Healthcare Life Sciences). Densitometry was performed using Image J software, and the results are presented as an average of 4 replicates.

### Western blot analysis

Proteins (20 μg per lane) were separated by SDS-polyacrylamide gel electrophoresis and were transferred onto nitrocellulose membranes. The membranes were blocked in 5% dry milk, incubated with the appropriate primary antibody solutions (5% BSA in PBS), followed by incubation with appropriate secondary antibody and immunodetected using the enhanced chemiluminescence method.

### Insulin secretion

Min6 cells were cultured at a concentration of 10^5^ cells/ml. After 48 h, cells were treated with leaf, fruit, and root extracts for 40 min. Cells were preincubated in KRBH buffer containing 25 mM NaHCO_3_, 115 mM NaCl, 4.7 mM KCl, 2.56 mM CaCl_2_, 1.2 mM MgSO_4_, 20 mM HEPES, 0.1% BSA and 2.8 mM glucose for 30 min and the supernatant was collected. The cells were then incubated in KRBH buffer containing 16.8 mM glucose for an additional 30 min. The supernatant was collected, and insulin concentration at the basal state (low glucose, 2.8 mM) and after induction (high glucose, 16.8 mM) was measured using an insulin immunoassay kit (Mercodia) according to the instructions of the manufacturer.

### Statistical analysis

Values are presented as means ± SEM. Statistical differences between the treatments and controls were tested by unpaired two-tailed Student’s *t*-test or one-way analysis of variance (ANOVA), followed by Bonferroni’s post-hoc testing when appropriate. Analysis was performed using the GraphPad Prism 5.0 software. A difference of *p* < 0.05 or less in the mean values was considered statistically significant.

## Results

### LDH release

**Fig. 1:**
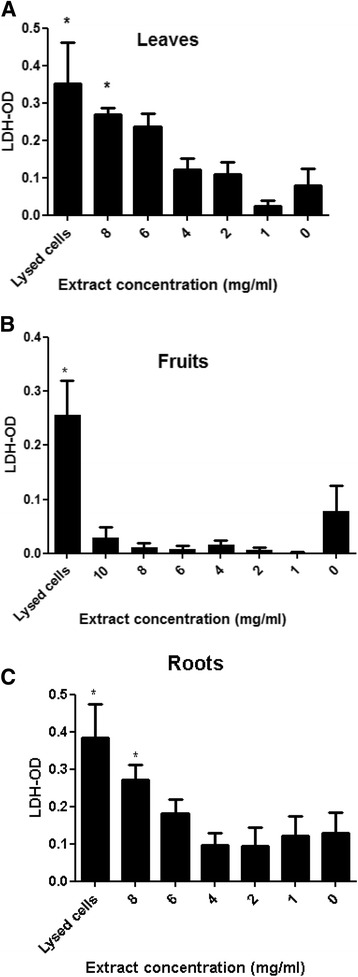
LDH release following treatment with *S. spinosum* extracts in 3 T3-L1 preadipocytes. Cells were cultured on a 96-well plate, at a concentration of 1 × 10^5^ cells/ml in the absence or presence of different concentrations of *S. spinosum* extracts (**a**. leaves, **b**. fruits, **c** roots). LDH was measured in the culture media as described in the *Materials and Methods*. Results are mean ± SEM of 3 independent experiments. *p < 0.05, **p < 0.005, ***p < 0.0005 compared to untreated cells by Student’s *t*-test

In order to identify the dose-range for the study, the dose-response cytotoxicity effect of the various extracts was measured. 3 T3-L1 pre-adipocytes were treated with extracts of *S. spinosum* for 24 h. LDH release was measured as a biomarker for cytotoxicity, demonstrating the complete absence of cell death in the presence of F/*S. spinosum* extract, at all concentrations measured, and a similar cytotoxic effect of L/*S. spinosum* and R/*S. spinosum* extracts at dose of 8 mg/ml (Fig. 1). Accordingly, the concentrations of extracts used in the next experiments were all in the non-toxic range.

### α-amylase and α-glucosidase inhibition

**Fig. 2:**
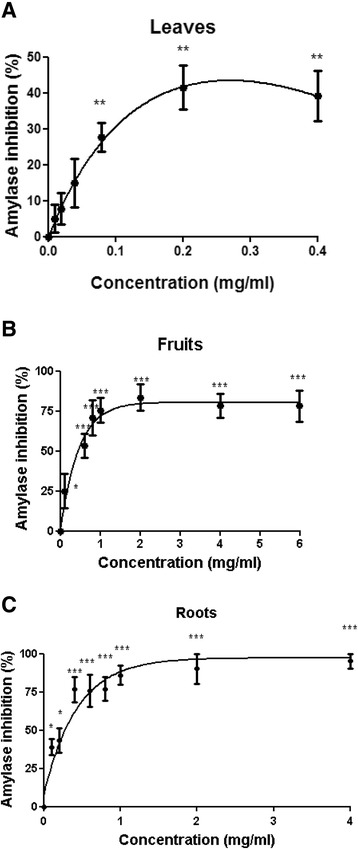
*S. spinosum* extracts inhibited the activity of α-amylase. The activity of α-amylase in the presence or absence of leaves (**a**), fruits **b** or roots **c** extracts was measured as described in *Materials and Methods*. Results are mean ± SEM of % inhibition of 5 independent experiments. *p < 0.05, **p < 0.005, ***p < 0.0005 compared to untreated cells by Student’s *t*-test

**Fig. 3:**
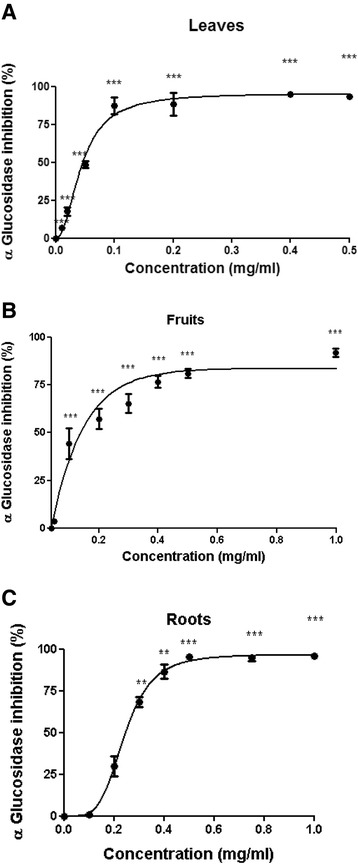
*S. spinosum* extracts inhibited the activity of α-glucosidase. The activity of α-glucosidase in the presence or absence of leaves (**a**), fruits **b** or roots **c** extracts was measured as described in *Materials and Methods*. Results are mean ± SEM of % inhibition of 5 independent experiments. *p < 0.05, **p < 0.005, ***p < 0.0005 compared to untreated cells by Student’s *t*-test

The capability of the extracts to inhibit α-amylase (Fig. 2) and α-glucosidase (Fig. 3) activity was measured. Both extracts of roots and aerial parts of *S. spinosum* inhibited the activity of α-amylase; however the Vmax for R/*S. spinosum* and F/*S. spinosum* extracts was much higher than that of L/*S. spinosum* extract (Table 1). On the other hand, all extract had a prominent inhibitory effect on α-glucosidase activity (Table 1).

**Table 1:** Vmax and IC50 values of various organs of *S. spinosum* against α-amylase and α-glucosidase activity

	α-amylase inhibition		α-glucosidase inhibition	
	Vmax	IC50 (mg/ml)	Vmax	IC50 (mg/ml)
Roots	98.35	0.16	96.48	0.21
Leaves	45.03	-	95.48	0.048
Fruits	71.22	0.29	85.32	0.125

### Glucose uptake

**Fig. 4:**
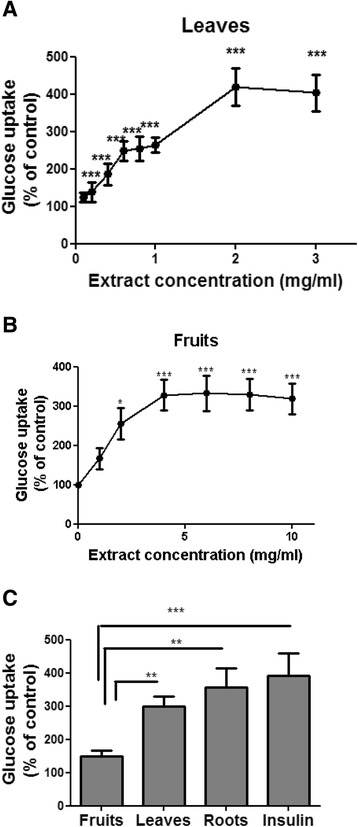
*S. spinosum* root extract is more effective than leaves or fruits extract in the induction of glucose uptake. Differentiated 3 T3-L1 adipocytes were treated with leaves **a** or fruits **b** *S. spinosum* extracts at various concentrations or all at 1 mg/ml, with the use of roots extract and insulin (100 nM) as positive control **c** for 30 min. The uptake of [3H]2-Deoxy-D-glucose into cells was determined as described in *Material and Methods*. Data are expressed as percent of basal uptake in control cells. The data represents the Mean ± SEM of measurement made on 3 replicates in each of at least 5 independent experiments. *p < 0.05, **p < 0.005, ***p < 0.0005 compared to untreated cells by Student’s *t*-test

The effect of extracts prepared from the aerial parts of the shrub on glucose uptake in 3 T3-L1 adipocytes is presented in Fig. 4. Glucose uptake was elevated in the presence of both L/*S. spinosum* and F/*S. spinosum* extract (Fig. 4a and b), however R/*S. spinosum* and L/*S. spinosum* extracts had higher potency than that of F/*S. spinosum* extract to induce glucose uptake, which is similar to the effect of insulin (Fig. 4c).

**Fig. 5:**
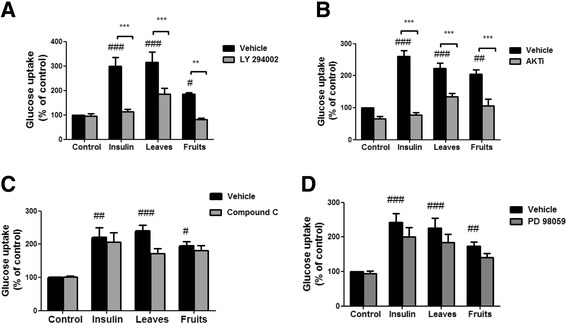
Glucose uptake induced by aerial parts of *S. spinosum* is mediated by PI3K and Akt. Differentiated 3 T3-L1 adipocytes were treated with leaves and fruits (2 mg/ml) or roots (1 mg/ml) extracts, with the use of insulin (100 nM) as a positive control, in the presence or absence of LY294002 (**a**), AKT inhibitor VIII (**b**), compound C (**c**), or PD 98059 (**d**). The uptake of [3H]2-Deoxy-D-glucose into cells was determined as described in *Materials and Methods*. Data are expressed as percent of basal uptake in control cells. The data represents the mean ± SE of measurement made on three replicates in each of at least 4 independent experiments. #*p* < 0.05, ##*p* < 0.005, ### *p* < 0.0005 compared to untreated cells, **p < 0.005, *** *p* < 0.0005 comparing cells treated with the indicated extract in the presence or absence of the indicated inhibitor, analyzed by one-way analysis of variance (ANOVA), followed by Bonferroni’s post-hoc testing

In order to clarify the pathway mediating the induction of glucose uptake by the aerial parts of *S. spinosum*, glucose uptake was measured in the presence or absence of specific inhibitors of PI3K, Akt, AMPK and ERK. L/*S. spinosum* and F/*S. spinosum*-induced glucose uptake was abrogated in the presence of PI3K and Akt inhibitors (Fig. 5a and b, respectively), which blocked insulin-induced glucose uptake as well. Inhibition of AMPK using its specific inhibitor, compound C, did not affect *S. spinosum*-dependent glucose uptake (Fig. 5c), neither did PD 98059, an inhibitor of the MAPK pathway and ERK activity (Fig. 5d).

### Phosphorylation of signaling proteins

**Fig. 6:**
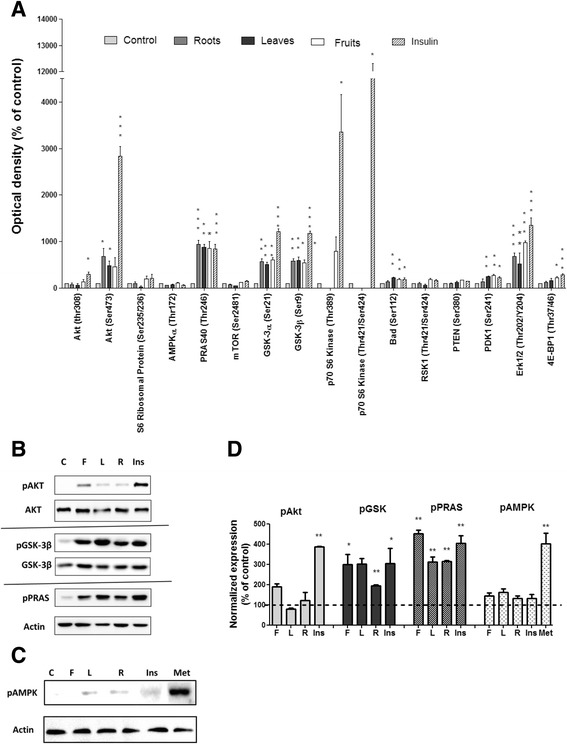
*S. spinosum* extracts induced the phosphorylation of Akt downstream protein with only minor phosphorylation of Akt. L6 myotubes were treated with 100 nM insulin or *S. spinosum* extracts for 20 min. **a** A simultaneous detection of 16 phosphorylated proteins predominantly belonging to the insulin signaling network was performed on whole cell lysates using PathScan analysis antibody array, as described in *Materials and Methods*. The results were detected by enhanced chemiluminescence and optical density was measured. Data are expressed as percent of OD obtained in control. The data represents the Mean ± SEM of 4 replicates. **b** and **c** L6 myotubes were untreated for negative control (C), or treated with 100 nM insulin (Ins), *S. spinosum* fruits (F), leaves (L) or roots (R) extracts or metformin (Met) for 20 min. Western-blot analysis of whole lysate was performed using specific antibodies. These are representative results of 3 independent experiments. The bar graph in **d** is the result of optical density measurements of Western blots in **b** and **c**. *p < 0.05, **p < 0.005, ***p < 0.0005 compared to untreated cells by Student’s *t*-test

In order to support these results, phosphorylation of key proteins involved in the induction of glucose uptake was measured in L6 myotubes in the presence of root, leaf and fruit *S. spinosum* extracts and insulin, used as a positive control. Phosphorylation of 14 different proteins on a total of 16 phosphorylation sites was measured by PathScan array (Fig. 6a). PathScan analysis demonstrated that insulin significantly increased the phosphorylation of 7 sites out of 16 measured; Akt (ser473), PRAS40 (thr246), GSK3α (ser21), GSK3β (ser9), PTEN (ser380), ERK1/2 (thr202/tyr204) and 4E-BP1 (thr37/46). However, while *S. spinosum* extracts increased the phosphorylation of some downstream proteins of Akt (PRAS40, GSK3β, PTEN and ERK1/2), the absence of Akt phosphorylation (ser473) following treatment with *S. spinosum* extracts is prominent. The effects of aerial and root extracts on protein phosphorylation were similar in most cases, except for PTEN which was not phosphorylated by root extract and 4E-BP1 which was phosphorylated only by F/*S. spinosum*. Neither insulin nor *S. spinosum* extracts increase the phosphorylation of AMPK on thr172.

Some of the results obtained by PathScan analysis were further validated by Western blot analysis (Fig. 6b), demonstrating that despite the presence of only minor phosphorylation of Akt, the phosphorylation of PRAS40 and GSK3β is highly induced by *S. spinosum* extracts. In addition, phosphorylation of AMPK was not observed following treatment by insulin or *S. spinosum* extracts (Fig. 6c).

### Insulin secretion

**Fig. 7:**
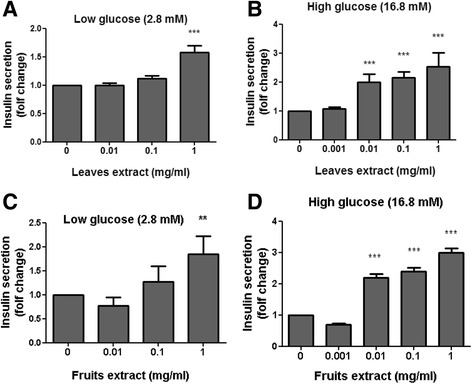
*S. spinosum* extracts induced insulin secretion in Min6 cells. Min6 pancreatic beta-cells were grown as described and treated for 1 h with leaves (**a** and **b**) or fruits (**c** and **d**) *S. spinosum* extracts at various concentrations as indicated. Supernatant was collected at the basal state and after glucose induction of insulin secretion. The induction of insulin secretion and measurement of insulin secretion were performed as described in the *Materials and Methods* section. Results are mean ± SEM of five independent experiments, **p < 0.005, ***p < 0.0005 compared to untreated cells by Student’s *t*-test

Other important targets of antidiabetic agents are the pancreatic insulin secreting β-cells. Induction of insulin release was measured under low and high glucose concentrations (Fig. 7) using Min6 cells, demonstrating that both L/*S. spinosum* and F/*S. spinosum* extracts increased insulin release, mainly under conditions of high glucose levels.

### HPLC and polyphenols analysis

**Fig. 8:**
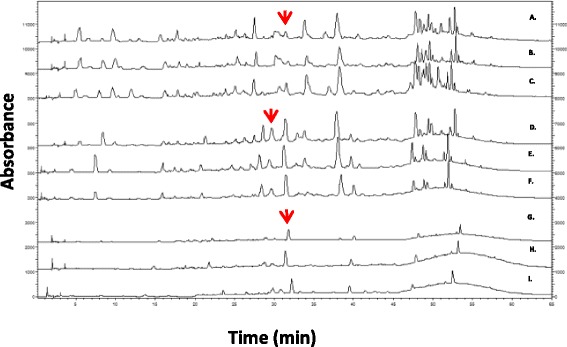
A representative RP-LC chromatograms of different extracts prepared from different plants. Leaves (A-C chromatograms), fruits (D-F chromatograms) and roots (G-I chromatograms) of *S. spinosum* (150 μg/run) recorded at 224 nm

Assaying the composition of phytochemicals in leaves and fruits of *S. spinosum* using RPLC revealed that catechin is present in all samples (Mean values are presented in Table 2). The levels change with relation to the date of harvest (all harvested during April-June). The levels of polyphenols in extracts from *S. spinosum* were evaluated using the Folin Ciocalteou method [26]. Results ranged in both organs from 100 mg to 376 mg/g dry weight of powder, depending on the date of harvest. RPLC profiles of 9 extracts (3 different independent extracts were prepared from root, leaf and fruit of *S. spinosum*) are presented in Fig. 8. Interestingly, the RPLC profile of the compounds is similar for all leaf and fruit samples, and one can easy differentiate leaves from fruits or roots based on this profile.

**Table 2:** Catechin and phenolics content in *S. spinosum* roots, leaves and fruits extracts (Average ± SD)

	Catechin (μg/mg)	Phenols (μg/mg)
Roots	16.5 ± 1.9	288.8 ± 45.6
Leaves	10.1 ± 4.5	126.4 ± 21.4
Fruits	14.9 ± 1.8	191.3 ± 70.1

## Discussion

Type 2 diabetes (T2D) is a complicated metabolic disease, developed as a result of genetic susceptibility and the presence of some environmental factors. More than 80 different loci associated with T2D have been identified, with inter-individual variation in the specific genetic architecture contributing to the development of the disease [28, 29]. This genetic variation leads to various pathogenic processes resulting in impaired glucose homeostasis, reflected in differing responses to medications among diabetic individuals. This variation emphasizes the need for anti-diabetic drug development, acting via diverse mechanisms, which might improve glycemic control of patients who are not well-balanced by already approved drugs. Utilizing traditional medicine, the search for new antidiabetic bioactive molecules may yield novel antidiabetic drugs.

*S. spinosum* has been recognized since ancient times, mentioned by Hippocrates and others, for its medicinal qualities, and has been used for many years in traditional Bedouin medicine for the treatment of diabetes [5–7, 16]. Most sources suggest the use of the root as the raw material for remedy in the treatment of diabetes [5, 6, 13], while the aerial organs are suggested for other purposes [9]. Although some medicinal plants demonstrate similar therapeutic properties with various organs of the same plant, in other plants this is not the case. In fact, one part of the plant may exert a beneficial medicinal property, while other parts of the same plant may be ineffective or even toxic [24, 30]. Thus, the presence of anti-diabetic functions of *S. spinosum* root-extract does not guarantee the presence of similar properties in other parts of the shrub. This study demonstrates for the first time that anti-diabetic effects are exerted by aerial organs of *S. spinosum,* in addition to the roots. The promising results obtained in this study regarding the beneficial glucose lowering properties of *S. spinosum* fruit and leaf should be cautiously implemented. Safety issues should be investigated in depth, as traditional medicine rarely uses the aerial parts of *S. spinosum*.

**Fig. 9:**
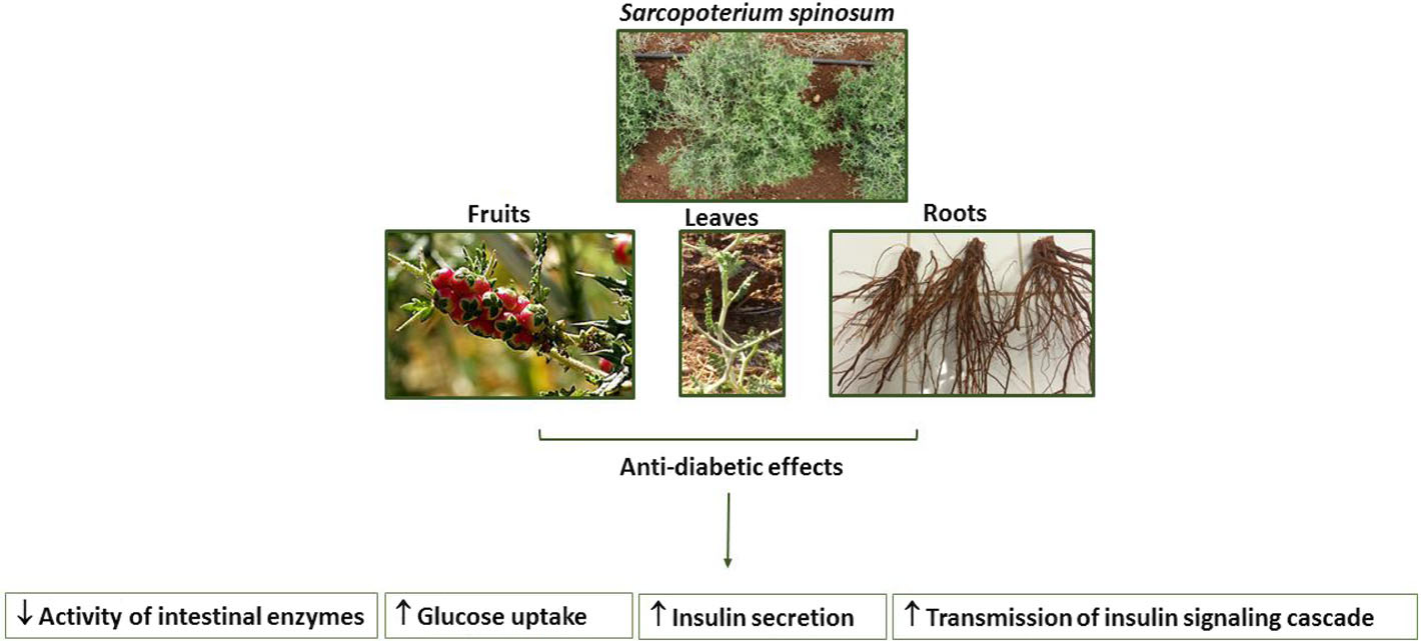
A schematic diagram of antidiabetic properties of extracts of various *S. spinosum* organs

An optimal regulation of blood glucose depends on the coordinated function of several different tissues and organs. These include the gut, responsible for carbohydrate digestion and absorption and pancreatic β-cells, responsible for accurate insulin secretion. Target tissues of insulin, such as the liver, muscle and adipose tissue, as well as the brain, are also involved in glucose regulation, responding to insulin by diverse pathways. This leads to an enhancement in glucose uptake, metabolism, and regulation of whole body energy balance. Accordingly, all of these functions may be the target of glucose lowering agents. Medicinal plants generally contain several bioactive molecules, producing different physiological effects. These act in concert in various complicated interactions to achieve their therapeutic effects. As such, this study demonstrated that *S. spinosum* affects several different processes (Fig. 9). It inhibited carbohydrate digestion, enhanced insulin secretion, and improved the transmission of insulin signaling in adipocyte and myotubes, either as an insulin sensitizer or as insulin mimetic agent, leading to the induction of glucose uptake.

*S. spinosum* extracts inhibited the activity of α-amylase and α-glucosidase, catalyzing the endo-hydrolysis of 1,4-α-D-glucosidic linkages in polysaccharides or the terminal-hydrolysis of 1,4-α-D-glucosidic linkages in oligosaccharides, respectively. Slowing carbohydrate digestion leads to a lower absorption rate, prevents glucose exaggerations in blood, and reduces the requirement for rapid insulin secretion. In addition, it is suggested that secretion of gut hormones, such as incretins, might be affected by slowing carbohydrate digestion [31].

Mechanisms of action, mediating the induction of glucose uptake by the extracts, seem to be similar between different organs of the plant. In our previous study, we demonstrated that Akt is not phosphorylated on ser473, a phosphorylation site recognized as required for the activation of this kinase, following treatment with root extract of *S. spinosum*. On the other hand, Akt was found to be activated by *S. spinosum*, as found by the membranal/nuclear translocation of this protein, and the phosphorylation of its downstream proteins [12]. These results indicate the presence of a unique mode of Akt activation by *S. spinosum* root extract. In this study we found that aerial organs of *S. spinosum* demonstrate similar effects. While the phosphorylation of Akt (ser473) is negligible, the phosphorylation of its effectors, PRAS40 (proline-rich Akt substrate 40) and GSK3β, is significantly induced. In addition, Akt is required for *S. spinosum*-dependent glucose uptake as demonstrated by the complete amelioration of the induction of glucose uptake by aerial organs in the presence of Akt inhibitor.

PRAS40 is known to be a target of Akt [32] and is highly phosphorylated (thr246) by *S. spinosum* extracts. Phosphorylation of PRAS40 disrupts the binding between PRAS40 and mTORC1. This enables the activation of mTORC1, which is associated with increased insulin sensitivity [33]. It was also postulated that there is another PRAS40-induced activity, yet to be identified, mediating the effect of PRAS40 as an enhancer of glucose uptake and regulator of insulin sensitivity, independent of mTORC1 pathway [34]. The role of PRAS40 in *S. spinosum*-dependent glucose uptake is currently under investigation.

ERK phosphorylation was increased by *S. spinosum* as well. However, this protein is not involved in the induction of glucose uptake, as measured in this study, representing acute effect of the extracts. The possible contribution of ERK to chronic effects of *S. spinosum* on insulin sensitivity should be investigated further.

Thus, our results indicate that *S. spinosum* enhanced glucose uptake by activating insulin signaling cascade, although upstream events leading to the activation of Akt, and the specific mechanism of Akt activation, are different from those recruited by insulin. In addition, as AMPK is not involved in the function of *S. spinosum*, it can be concluded that the mechanism of action of the extracts is different from that of metformin, which is the commonly used antidiabetic medication. We conclude that active ingredients in *S. spinosum* facilitate glucose uptake by utilizing a unique mechanism. Clarifying its mechanism of action may lead to the development of new agents for the treatment of insulin resistance that might improve glycemic control in patients that do not adequately respond to common anti-diabetic medications and might benefit from a drug acting on a different molecular target.

## Conclusion

This study demonstrated the anti-diabetic properties of the various organs of *S. spinosum* and emphasizes the importance of identifying the active ingredients in *S. spinosum* in order to enable the development of dietary supplements or new drugs for the treatment of diabetes. As most ethno-pharmacological documentation indicates the use of *S. spinosum* root as an antidiabetic remedy, caution must be used and the whole battery of toxicological studies performed to ensure that no safety concerns exist associated with the use of extracts prepared from the aerial parts of *S. spinosum*.

## Abbreviations

2-DG: 2-deoxy-d-glucose
AMPK: AMP -activated protein kinase
ERK: Extracellular signal–regulated kinases
GSK3β: glycogen synthase kinase 3β
LDH: lactate dehydrogenase
mTORC1: mammalian target of rapamycin complex 1
PI3K: Phosphatidylinositide 3-kinase
PRAS40: proline-rich Akt substrate of 40 kDa
PTEN: phosphatase and tensin homolog
*S. spinosum* fruit: F/*S. spinosum*
*S. spinosum* leaves: L/*S. spinosum*
*S. spinosum* root: R/*S. spinosum*
*Sarcopoterium spinosum*: *S. spinosum*
T2D: Type 2 diabetes
